# Anti-diabetic Potential of Indigenous Medicinal Plants of Cholistan Desert, Pakistan: A Review

**DOI:** 10.1900/RDS.2022.18.93

**Published:** 2022-06-30

**Authors:** Tahira Shamim, Hafiz Muhammad Asif, Ghazala Shaheen, Laila Sumreen, Sultan Ayaz, Tasneem Qureshi, Aymen Owais Ghauri, Tanveer Ali, Mukhtiar Ahmad, Farhan Sajid, Ijaz Khadim, Rida Tanveer, Raeesa Noor, Hina Nawaz, Jahanzaib Kaleem

**Affiliations:** 1 Department of Eastern Medicine & Surgery, University College of Conventional Medicine, The Islamia University of Bahawalpur, Pakistan,; 2 Department of Homoeopathic Medical Sciences, University College of Conventional Medicine, The Islamia University of Bahawalpur, Pakistan,; 3 Department of Eastern Medicine, Faculty of Medical Science, Government College University, Faisalabad, Pakistan,; 4 Faculty of Eastern Medicine and Natural Sciences, Ziauddin University Karachi, Pakistan,; 5 Department of Eastern Medicine, Faculty of Allied Health Sciences, Jinnah University for Women, Karachi, Pakistan,; 6 Department of Library & Information Science, Faculty of Social Sciences, The Islamia University of Bahawalpur, Pakistan,; 7 AGP Pharmaceutical Pvt- Ltd, Karachi, Pakistan.

**Keywords:** cholistan desert, medicinal plants, anti-diabetic, diabetes mellitus

## Abstract

Cholistan Desert is a sandy desert located in southern Punjab, Pakistan. The area is rich in more than 64 medicinal plants among 138 plant species. It is noteworthy that this remote desert lacks modern health care facilities and its inhabitants are dependent on locally-available plant species for the treatment of acute and chronic illnesses. Medicinal plants, traditionally have been ideal sources of remedies for the management of many non-communicable diseases; most modern prescriptions drugs have their origins from plants. Diabetes is increasing at an alarming rate in the past few decades. Whereas medicinal plants are used globally, the specific properties of only a few have been identifies scientifically. Similarly, little scientific evidence exists that confirms the efficacy of the medicinal plants of this region for diabetes management. Ethnobotanical studies show that locally-available medicinal plants do have anti-diabetic potential. We reviewed the medicinal properties of 36 of these plants. Several ingredients derived from these plants have chemical constituents that demonstrate anti-diabetic activity, thereby validating their importance for the management of diabetes.

## 1 Introduction

Pakistan is blessed with diversified irrigated immense plains, elevated peaks, coasts, snow peaked mountains, and freezing and burning deserts [1]. The Cholistan Desert which is also known as Rohi is a large desert in the southern area of Punjab, Pakistan [2]. It encompasses an area of 26,000 km2 [3]. The climate of the Cholistan Desert is burning hot, sub-tropical, with less monsoon rainfall and accompanied by intermittent and long droughts, giving it arid, and especially strong summer winds that offer comparatively high evaporation rates and low humidity reflecting the unmatched wealth of its flora [4]. Medicinal plants are the basic element for the management of many illnesses and have significant importance in the life of its population [5]. These are also an important income source for poor field workers and people who are linked with the manufacturing of herbal products [6]. Conventional healthcare knowledge becomes shared between generations, usually verbally, rather than as a written document. It is the main reason that this inter-generational knowledge is diminishing [7]. Whereas many researchers have studied the ethnobotanical, medicinal and economic significance of plants, sufficient research has yet to be done [8]. However there have been a massive undertaking of research during the past few years in a several institutions to examine the anti-oxidant, antimicrobial, anti-inflammatory, anti-cancer efficacy of the medicinal plants [9].

Diabetes mellitus is not only a complex disease, but also a chronic metabolic disorder having hyperglycemia, glycosuria and negative nitrogen balance as its main features [10]. Diabetes occurs because of a decrease in insulin secretion by beta cells of the pancreas and poor response of insulin receptors to insulin [11]. Currently, diabetes is a prevalent disease that i affecting the 25% of world’s adult population. It affects around 150 million people and this number is about to reach 300 million by 2025 [12]. Diabetes is a disease that is still not completely curable through currently available anti-diabetic drugs [13]. In developing countries, like Pakistan, these products are not easily available and are also high-priced [14]. The key defect in carbohydrate metabolism in diabetes results in extensive, multi-organ complications that eventually involve every system of the body [15]. It also is true that with the advancement of knowledge about the pathophysiology of diabetes mellitus, and better understanding of its development, this syndrome in now at the front line of research in molecular biology and immunology [16]. Because diabetic complications are a serious health issue and can worsen with every passing day, there is need for an efficacious treatment for its management and prevention of complications [17].

Presently, available therapies for the management of diabetes include insulin and oral anti-diabetic agents such as biguanides, sulfonylurea, glinides and α-glucosidase inhibitors [18]. Despite the launch of metformin and sulfonylurea about 50 years ago, no considerable lead has been achieved for better management of diabetes [19]. This is the failure of synthetic drugs and a question mark on authorities addressing the management of diabetes mellitus [20]. Many plant preparations are being prescribed by conventional healers and these preparations also are accepted by patients experiencing diabetes and many other illnesses throughout the world, especially in third-world countries [21]. Therefore, a thorough scientific investigation of, and research about medicinal plants by chemical investigation, followed by pharmacological screening, is warranted [22]. Plant extracts and plant preparations have remarkable effectiveness in treating diabetes and its complication with less notable side effects [23]. Currently, whereas there are numerous traditional medications on the market, science still seeks the best medicine for the prevention and management diabetes mellitus. The purpose of this review was to summarize the research base about indigenous medicinal plants of the Cholistan Desert, Punjab, Pakistan so that they can be further investigated for their specified anti-diabetic properties along with their mechanism of action.

### 1.1 Classification of anti-diabetic drugs

Many herbal preparations and herbal extracts have the capacity to treat diabetes mellitus. Their actions categorize as described in the section below:

Extracts/drugs acting as α-glycosidase or α-amylase inhibitor

Drugs/extracts in this category have the capacity to lower blood glucose level by inhibiting gastric enzymes (α-glycosidase or α-amylase) that are compulsory for the breakdown of polysaccharides to simple sugar. Several medicinal plants have the potential to inhibit the α-glycosidase and α-amylase activity and can be employed for the treatment of both Type I and Type II diabetes [24].

Methanolic leaf extract of *Adhatoda vasica* (Nees.), of the family Acanthaceae, shows the highest α-glycosidase or α-amylase inhibitory activity [25]. Enzyme-guided fractionation of leaf extract of *A. vasica* was followed by the isolation vasicine and vasicinol as intestinal enzyme inhibitors [26].

Extracts/drugs increases insulin secretion or β-cell regeneration

Drugs in this category are linked with Type I or IDDM diabetes in which there is decrease in the number of β islet cells of Langerhans leading to relative or absolute deficiency of insulin.

Ethyl acetate extract of radix of *Acorus calamus* is used to sensitize the insulin activity [27]. Ginsenoside Rh2 is the active constituent found in *Panax ginseng* root that increases blood insulin levels along with decreasing blood glucose levels [28].

Extracts/drugs act as hypoglycemic, anti-hyperglycemic or anti-diabetic effect

Herbal drugs in this category can decrease blood glucose levels directly and applied to the treatment of both IDDM and NIDDM types of diabetes mellitus.

*Abroma augusta* (locally known as Ult kambal) leaf extract shows hypoglycemic effect on alloxan induced diabetic rats [29]. *Acacia arabica* (Fabeceae) bark exhibits antihyperglycemic effect in streptozotocin-induced diabetic rats [30].

Extracts/drugs dealing with the complications of diabetes mellitus

Diabetes mellitus is a chronic metabolic disorder characterized by alteration in carbohydrate metabolism that is associated with reduction in insulin secretion or its action by which blood glucose level increases [31]. This uncontrolled hyperglycemia can lead to the onset of number of complications in patients with diabetes [32]. Herbal extracts/preparations can be used as an effective remedy for the treatment of these complications [33].

*Moringa oleifera* Lam. seed powder was investigated by Abdulrahman and Haddad for its anti-diabetic effect and for the treatment of diabetic neuropathy in streptozotocin-induced diabetic male rats [34].

## 2 Methods

### 2.1 Literature search process

This review is based on ethno-botanical and pharamcological evidences-based research of the medicinal plants from the Cholistan Desert for the management of diabetes mellitus. We searched the literature using the keywords anti-diabetic, and medicinal plants in the indices Pub Med, Science Direct, Google Scholar, Scopus, Web of Science, Wiley Online Library, and Medline and published monographs, reports and articles etc. We searched several articles and other relevant information; we screened the data for this study with respect to anti-diabetic potential of indigenous medicinal plants of the Cholistan Desert.

## 3 Results

### 3.1 Ethno-botanical and pharmacological data

There is a list of medicinal plants evaluated scientifically based on ethno-botanical knowledge. All identified medicinal plants have received study on diabetic models and are undergoing trials. Some of the data for these medicinal plants appear in Table 1.

**Table 1.** Ethno-botanical and pharmacological data of medicinal plants from cholistan desert **Sr**.

Sr. No.	Botanical name	Common name	Family	Parts used	Effects observed	References
1	*Abroma augusta*	Ult kambal	*Sterculiaceae*	Roots, Leaves	Hypoglycemic, antidiabetic	[35], [36]
2	*Acacia arabica*	Kekar, Babool	*Fabeceae*	Bark, Pods, Seeds,	Antidiabetic	[37] [38]
3	*Azadirachta indica*	Neem	*Meliaceae*	Leaves, Flower, Seed, Root bark	Antidiabetic	[39, 40]
4	*Borerhavia diffusa*		*Nyctaginaceae*	Whole plant	Antidiabetic	[41]
5	*Capparis deciduas*	Delha, Kair	*Capparaceae*	Fruit, Stem,	Antidiabetic	[42, 43]
6	*Gymnema sylvestre*	Gurmaar boti	*Asclepiadaceae*	Leaves/Callus/Stem	Antidiabetic, Hypoglycemic,	[44-46]
7	*Momordica charantia*	Karela	*Cucurbitaceae*	Seeds	Antidiabetic	[47]
8	*Silybum marianum*	Onsst kattara	*Asteraceae*	Seeds	Antidiabetic	[48, 49]
9	*Tribulus terrestris*		*Zygophyllaceae*	Aerial Part, Fruit	Antidiabetic, Anti-hyperglycaemic, Hypoglycemic	[50-52]
10	Adhatoda vasica	Adosa, Bansa	*Acanthaceae*	Leaves	α-glucosidase inhibition	[26]
11	Allium cepa	Piyaz	*Amaryllidaceae*	Bulb, Leaves, Seeds	Antidiabetic, Hypoglycemic	[53, 54]
12	Allium sativum	Lehsan	*Amaryllidaceae*	Bulb, Leaves, Seeds	Antidiabetic, Hypoglycemic	[55-57]
13	Aloe vera	Kanwar gandal	*Liliaceae*	Whole plant	Antidiabetic	[58-62]
15	Cajanus cajan	Arhar	*Fabaceae*	Leaves, Seeds	Antidiabetic, Hypoglycemic	[63-65]
16	Caralluma edulis		*Apocynaceae*	Roots	Antidiabetic, Hypoglycemic	[38]
17	*Citrullus colocynthis*	Kor tumma	*Cucurbitaceae*	Roots, Pulp, Seeds	Antidiabetic, Hypoglycemic	[66-68]
20	Corchorus depressus	Bahupali	*Malvaceae*	Aerial parts and roots	α-glucosidase inhibiton	[69, 70]
21	*Cyperus rotundus*	Nagarmotha	*Cyperaceae*	Rhizomes	α-glucosidase & α-amylase inhibiton, antidiabetic activity	[71-73]
22	*Euphorbia hirta*	Dudhi	*Euphorbiaceae*	Leaf, Flower and stem	antidiabetic activity	[74-77]
23	*Farsetia hamiltonii*	Fareed boti	*Brassicaceae*	Aerial parts	antidiabetic activity	[78]
25	*Salvadora oleoides*	Pilu	*Salvadoraceae*	Aerial part	antidiabetic activity	[79]
27	*Withania somnifera*	Ashwagandha	*Solanaceae*	Leaf and root	antidiabetic activity	[80]
28	*Ziziphus nummularia*	Ber	*Rhamnaceae*	Leaves	antidiabetic activity	[81, 82]
30	*Aerva javanica*	Sparai	*Amaranthaceae*	Aerial parts	antidiabetic activity	[83]
32	*Suaeda fruticosa*		*Chenopodiaceae*	Aerial part	hypoglycemic activity	[84]
33	*Achyranthes aspera*	Chirchita	*Amaranthaceae*	Whole plant	Antidiabetic and hypoglycemic activity	[85-87]
35	*Alhagi maurorum*	Javasa	*Leguminosae*	Aerial parts	Antidiabetic	[88]
36	*Calotropis procera*	Aak	*Asclepiadaceae*	Aerial parts	Antidiabetic	[89-91]

## 4 Discussion

Diabetes mellitus is a syndrome whose occurrence is on the rise globally [92]. Despite discovery of many anti-diabetic remedies in the field of medicine, plant preparations are used extensively and effectively for the management of this illness [93]. Treatment of diabetes with conventional medicinal plants is in vogue throughout the globe [94]. Medicinal plant remedies are considered to have fewer adverse side effects and less toxicity compared to synthetic drugs [95]. The anti-hyperglycemic effects shown by these plants are due to their capability of increasing insulin synthesis, and decreasing glucose absorption from intestines or regulation of the pancreatic function [96]. Although herbal preparations have protective effects on β-cells and help in regulating blood glucose levels, scientific understanding of the mechanism of action for these plants is lacking [97]. Many plants have active constituents such as alkaloids, flavonoids, glycosides, tannins, terpenoids, resins, saponins and other properties that are used because of their anti-diabetic effects [98]. Berberine is an alkaloid with anti-diabetic properties, it decreases transport glucose through the intestinal epithelium by inhibiting alpha-glucosidase [99]. Imidazoline causes the increased secretion of insulin in a glucose-dependent manner [100]. Polysaccharides from pumpkin show anti-hyperglycemic activity by lowering the blood glucose level, by stimulating insulin secretion and by improving glucose tolerance [101]. Quercetin is a flavonoid that reduces the level of glucose with a significant reduction of plasma triglyceride and cholesterol [102]. It also enhances the activity of hepatic glucokinase by stimulating the release of insulin from β-cells of pancreas [103]. Fractions rich in insoluble fiber have been isolated from *Citrus sinensis* peel and used to decrease glucose diffusion and decrease the rate of glucose absorption by inhibiting alpha-amylase [104]. Steroidal glycosides and triterpenoid (saponin) increase the concentration of insulin in blood and block the synthesis of glucose [105]. Ferulic acid isolated from rice bran has been effective in stimulating the secretion of insulin [106].

**Figure 1.**
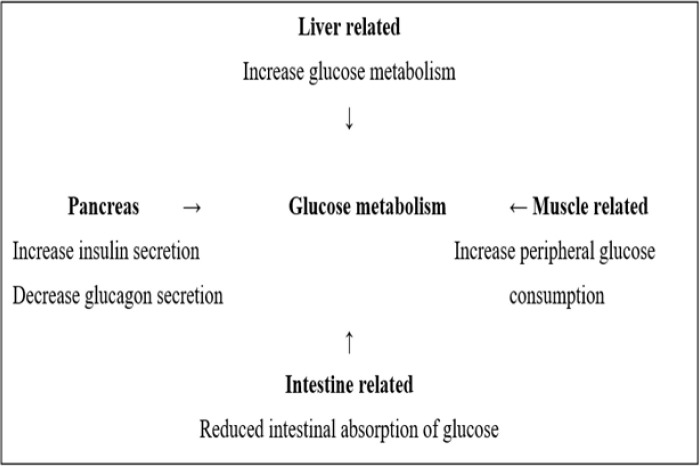
Mechanism of glucose metabolism.

Employment of whole plant, leaves, roots, bark, fruit, seeds, flower, gum and rhizome etc. of different plant species predispose these medicinal plants to a high degree of threat. Medicinal plants receive use in conventional systems of medicine, as well as by plant-based pharmaceutical industries and are accessible in their natural habitats. If such practices continue, many plant species might decrease and eventually vanish from their natural domain. This is specifically true for medicinal plants with anti-diabetic potential. Therefore, there is need to encourage the community for cultivation of medicinal plants which can serve the basis of new medicinal products. Moreover, climate change as well as seasonal variation in this area provide suitable environment for medicinal plant cultivation, which will be beneficial for maintaining the plant diversity in the region, in addition to enhancing the socioeconomic lifestyle of the local population.

### 4.1 Conclusion

The medicinal plants identified in this paper have vital role in the prevention and management of diabetes mellitus. Although there is less knowledge about the mechanisms of action of the components found in these medicinal plants, there is no doubt about the importance of medicinal plants for disease management. Moreover, it is equally important that scientists screen the diversity of medicinal plants around the globe for bioactive phytomolecules that are potent against diabetes. Additionally, scientists should review conventional plant-based anti-diabetic remedies so that remedies can be available to people unable to buy expensive manufactured drugs. Consequently, plant preparations can play a role as an emerging substitute to currently used standard drugs to address the consequences of diabetes mellitus.

## Abbreviations

IDDM: Insulin-dependent diabetes mellitus

NIDDM: Non-insulin-dependent diabetes mellitus
